# Outbreeding effects in an inbreeding insect, *Cimex lectularius*

**DOI:** 10.1002/ece3.1373

**Published:** 2014-12-28

**Authors:** Toby Fountain, Roger K Butlin, Klaus Reinhardt, Oliver Otti

**Affiliations:** 1 Animal and Plant Sciences, University of Sheffield, Western BankSheffield, S10 2TN, UK; 2 Department of Biosciences, University of HelsinkiPO Box 65 (Viikinkaari 1), FI-00014, Helsinki, Finland; 3 Sven Lovén Centre – Tjärnö, University of GothenburgSE, 452 96, Strömstad, Sweden; 4 Institute for Evolution and Ecology, University of TübingenAuf der Morgenstelle 28, D-72076, Tübingen, Germany; 5 Animal Population Ecology, Animal Ecology I, Bayreuth Center of Ecology and Environmental Research (BayCEER), University of BayreuthUniversitätsstrasse 30, 95440, Bayreuth, Germany

**Keywords:** *Cimex lectularius*, colonization, inbreeding, metapopulation dynamics, outbreeding depression

## Abstract

In some species, populations with few founding individuals can be resilient to extreme inbreeding. Inbreeding seems to be the norm in the common bed bug, *Cimex lectularius,* a flightless insect that, nevertheless, can reach large deme sizes and persist successfully. However, bed bugs can also be dispersed passively by humans, exposing inbred populations to gene flow from genetically distant populations. The introduction of genetic variation through this outbreeding could lead to increased fitness (heterosis) or be costly by causing a loss of local adaptation or exposing genetic incompatibility between populations (outbreeding depression). Here, we addressed how inbreeding within demes and outbreeding between distant populations impact fitness over two generations in this re-emerging public health pest. We compared fitness traits of families that were inbred (mimicking reproduction following a founder event) or outbred (mimicking reproduction following a gene flow event). We found that outbreeding led to increased starvation resistance compared to inbred families, but this benefit was lost after two generations of outbreeding. No other fitness benefits of outbreeding were observed in either generation, including no differences in fecundity between the two treatments. Resilience to inbreeding is likely to result from the history of small founder events in the bed bug. Outbreeding benefits may only be detectable under stress and when heterozygosity is maximized without disruption of coadaptation. We discuss the consequences of these results both in terms of inbreeding and outbreeding in populations with genetic and spatial structuring, as well as for the recent resurgence of bed bug populations.

## Introduction

Most populations exist with some form of spatial structure due to subdivided habitat. Coupled with restricted dispersal, this subdivision can lead to metapopulation dynamics of frequent local extinctions and recolonization events between habitat patches. In turn, metapopulation dynamics can have dramatic consequences for the distribution and maintenance of genetic diversity and lead to fine-scale genetic structuring between subpopulations (Wade and McCauley 1988; Whitlock and McCauley 1990; Haag et al. 2005; Torimaru et al. 2007). Colonizing groups often consist of small numbers of individuals, and subsequent founder events can lead to a genetic bottleneck with substantial reductions in genetic variation (e.g., Haag et al. 2005). This genetic structure has important ecological and evolutionary implications, the most critical of which perhaps being increased inbreeding risk (Keller and Waller 2002; Bretman et al. 2011).

It is well established that inbreeding can be detrimental to individual fitness, known as inbreeding depression (Charlesworth and Charlesworth 1987; Keller and Waller 2002). Recessive deleterious alleles are maintained at low levels in populations through mutation–selection balance. Mating between relatives leads to an increase in genome-wide homozygosity and increased expression of deleterious recessives (the “dominance” effect) (Charlesworth and Willis 2009). An increase of homozygosity at loci with heterozygote advantage may also lead to a reduction in individual fitness (the “overdominance” effect) (Charlesworth and Willis 2009). As well as individual fitness effects, inbreeding has been shown to increase population extinction risk (Saccheri et al. 1998).

Population genetic theory suggests that, in species prone to inbreeding, deleterious alleles will be exposed to selection and purged from populations over time (Glémin 2003). While this purging may be effective for deleterious alleles with large effect (e.g., lethal recessives), deleterious alleles with smaller effects may be invisible to selection and drift to fixation (Keller and Waller 2002). Despite this, purging has been established as efficient under certain conditions, including when bottlenecks are very narrow (Glémin 2003; Facon et al. 2011). In particular, consanguineous mating has been shown to increase the efficiency of purging (Barrett and Charlesworth 1991; Wang 2000; Glémin 2003; Fox et al. 2008; Pujol et al. 2009). Purging therefore may play an important role in organisms that frequently go through genetic bottlenecks, such as those whose founding groups consist of small numbers of related individuals.

Populations may also recover from the detrimental effects of inbreeding through outbreeding. This can cause a positive shift in mean fitness through heterosis (Ingvarsson 2001). For example, there is evidence to suggest that the introduction of novel alleles through immigration may increase population growth rate in metapopulations (Ebert 2002; Gaggiotti et al. 2004; Haag et al. 2005). However, outbreeding does not always enhance fitness. Hybrid incompatibility in interspecies crosses is well documented, but crosses between distant populations of the same species can also be detrimental (Lynch 1991; Marr et al. 2002; Bomblies et al. 2007; Seidel et al. 2008; Whitlock et al. 2013). This outbreeding depression may be due to loss of local adaptation or a breakup of coadapted gene complexes independent of habitat (Lynch 1991; Charlesworth and Willis 2009). These effects may not be detected until the *F*_*2*_ (Whitlock et al. 2013) as recombination and segregation may only then separate allele combinations present in the parents (Lynch and Walsh 1998, p. 224), thereby reducing intrinsic coadaptation (Tallmon et al. 2004). Incompatible multilocus allele combinations may be recessive and thus only apparent after the generation of homozygotes in the *F*_2_ (Charlesworth and Willis 2009). Some structured populations have also been shown to have both inbreeding and local outbreeding depression (Sletvold et al. 2012), and interpopulation variation in response to inbreeding and outbreeding has been observed in metapopulations (Escobar et al. 2008). It is therefore important to understand the relative significance of inbreeding and outbreeding in populations with genetic and spatial structuring. While much work has been performed in understanding these dynamics in selfing hermaphrodites (Whitlock et al. 2013), much less attention has been paid to naturally inbreeding animals (but see Kureck et al. 2012; Berger-Tal et al. 2014).

**Figure 1:**
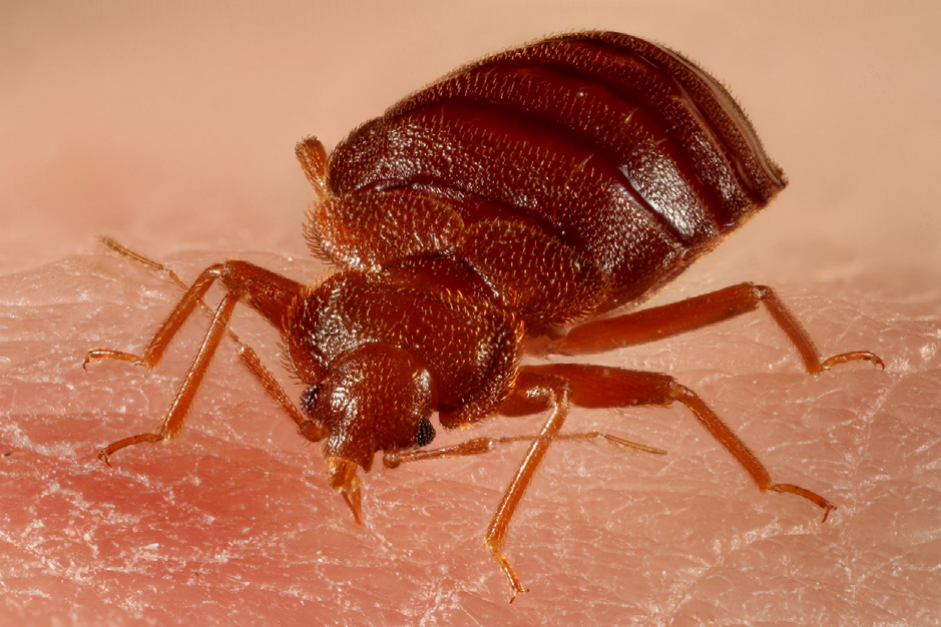
An adult common bed bug feeding on a human host (Photo Credit: Richard Naylor).

The common bed bug, *Cimex lectularius* L. (Fig.1)*,* is rapidly re-emerging as a prominent public health and economic pest because its populations can build up rapidly to large infestations and because new areas are rapidly colonized (Boase 2001; Doggett et al. 2004; Romero et al. 2007; Richards et al. 2009). Bed bugs are flightless and so can only move limited distances actively. Much of their recent success in spreading has been attributed to human-facilitated passive dispersal (Doggett et al. 2004). Due to founder events, frequent local extinctions caused by pest control, and restricted dispersal, bed bugs exist in highly structured metapopulations (Fountain et al. 2014). The number of founders has been estimated as being as low as a single-mated female, and with gene flow very low or absent between established demes, this results in very limited genetic diversity within infestations (Booth et al. 2012; Fountain et al. 2014). Inbreeding is therefore likely to be a very common feature of bed bug infestations. The small effective size of demes coupled with low gene flow results in very high levels of differentiation between infestations (Saenz et al. 2012; Fountain et al. 2014). However, multiple introductions into buildings may also occur (Booth et al. 2012), providing the opportunity for individuals from highly differentiated populations to meet, and hence the possibility of heterosis and/or outbreeding depression. While it is likely that bed bugs are resilient to inbreeding, what is not known is how outbreeding contributes to the successful establishment of populations. One hypothesis is that the increased connectivity between populations through an increase in global travel has led to a rise in multiple introductions into the same building (increasing outbreeding), which in turn has resulted in increased population growth and contributed to the bed bug's resurgence (Reinhardt 2012). In this study, we investigated the effect of outbreeding of bed bugs on four ecologically relevant fitness traits: fecundity, egg viability, body size of adult offspring, and starvation resistance in adult offspring.

## Materials and Methods

### Study species and general culture

Bed bugs were reared at 26°C and 70% relative humidity as previously described (Reinhardt et al. 2009). Virgin adults were produced by separating last instar nymphs from the stock populations and keeping them individually in 96-well plates provided with filter paper disks. On eclosion, they were sexed and isolated as in Reinhardt et al. (2009). Individuals from four stock populations, each with a different origin and demographic history (see Table1 for details), were used to assess whether populations responded to inbreeding differently. Stock populations were kept on a regime of 12 h light:12 h dark cycle in pots with filter paper and roughly 100 adults. To generate individuals for crossing trials, five family lines were set up using randomly selected virgin individuals, subsequently referred to as G_0_, for each stock population.

**Table 1:** Summary of stock populations used. For field-collected populations, the estimated number of establishing individuals is given. Mixed stock was established from an equal number of male and female individuals from both parental stocks. The estimated number of generations reared in the laboratory is also shown

Stock population	Origin	Establishing numbers	Generations
Lab Stock	London School of Hygiene and Tropical Medicine	Unknown	>300
Field UK	London, UK	200	25
Field Kenya	Near Nairobi, Kenya	15	10
Mixed Stock	Lab Stock x Field UK cross	Equal numbers of both parental stocks	2

### Crossing scheme

**Figure 2:**
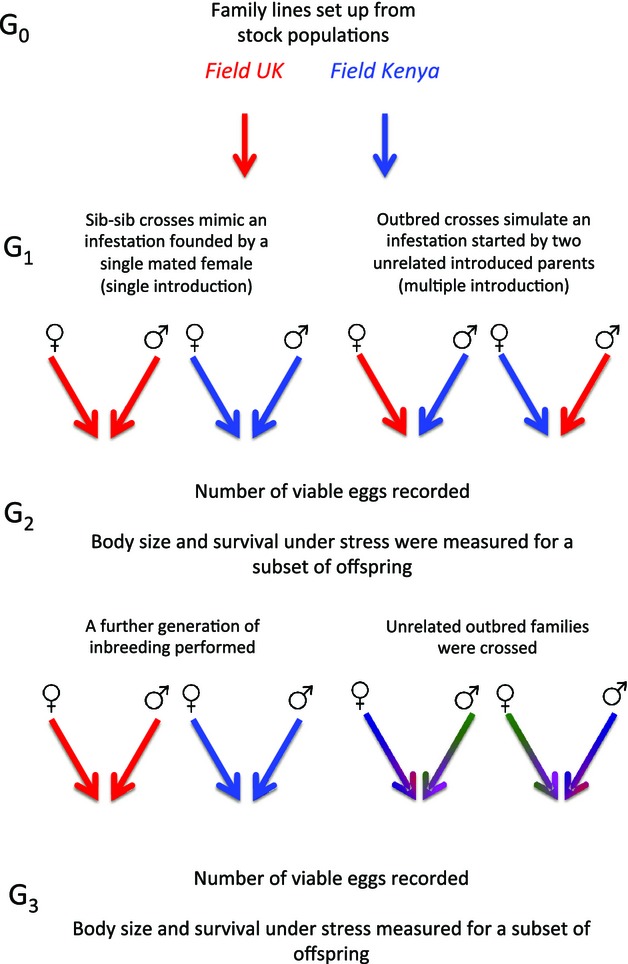
Example of experimental crossing scheme (simplified so that only two of the four stock populations are shown). Five family lines were set up for each stock population (G_0_). Offspring from these lines (G_1_) were used to set up 40 inbred families through sib–sib mating and 40 outbred families through between-population crosses (Table S1). Offspring from these lines (G_2_) were used to set up a further generation of inbreeding (once again sib-mated) and outbreeding (through crossing with unrelated G_2_ individuals, green and purple arrows represent an unrelated outcrossed individual).

The focus of this study was to test two realistic colonization scenarios of bed bugs. Therefore, two treatments were set up, sib–sib crosses and outbred (between population) crosses. Sib–sib crosses mimicked reproduction in infestations founded by a single sib-mated female (Fountain et al. 2014), while outbred crosses simulated reproduction in an infestation started by a female mated to an unrelated male (Booth et al. 2012) (Fig.2). Using the offspring of G_0_ lines (G_1_ individuals), 40 inbred families were created through sib–sib mating and 40 outbred families created through between-population crosses (Table S1). As G_0_ lines were not fully independent, when creating inbred families, we randomly selected two females and two males from each of the five lines per stock population. This resulted in 10 families per population and ensured a balanced design with no line overrepresented within a stock population. The outbred families included reciprocal crosses for each between-population combination (Table S1), ensuring any directional effect of outbred mating was accounted for. Each female was allowed to mate once for a standardized mating duration of 60 seconds (Reinhardt et al. 2009). Females were kept individually, provided with a strip of filter paper and fed ad libitum throughout the egg-laying period using the protocol of Hase (1930). Fitness measures (see below) were recorded for the offspring of these families (G_2_).

To examine the continued fitness effects of inbreeding and outbreeding, inbred and outbred families were maintained for another generation (Fig.2). G_2_ individuals from inbred families were once again sib-mated and outbred families were again outbred through crossing with unrelated G_2_ individuals (i.e., neither parent had a shared maternal or paternal population). Between-population crosses were performed by alternating the origin of the mother and the father so that each outbred reciprocal cross was performed a minimum of three times. This ensured that any directional effect of cross was taken into account. Egg counts were performed for each cross and fitness measures recorded for offspring (G_3_).

### Fitness measurements

We recorded two types of fitness measure, the number of viable eggs produced and the fitness of offspring (for details, see next paragraph). As this experiment is mimicking two colonization scenarios, the number of viable eggs produced by a cross is an ecologically relevant measure in the successful establishment and growth of populations. We therefore firstly performed egg counts to provide a measure of fecundity (Fig.2). Over an 8-week laying period, we counted weekly the number of eggs on the filter paper on which each female was kept. We recorded the number of viable and inviable eggs, the latter being easily distinguished by their brown color and the lack of red eyes normally visible through the eggshell in viable eggs (Reinhardt et al. 2009). Each week, females were provided with fresh filter papers. Egg counts were conducted until females stopped laying or were laying only inviable eggs for two consecutive weeks.

Secondly, we measured two offspring fitness traits to assess the effect of inbreeding and outbreeding on G_2_ and G_3_ individuals (Fig.2); adult survival under stress; and body size. As heterosis may be more pronounced under stressful environmental conditions (Armbruster et al. 1997; Armbruster and Reed 2005), we measured the survival of adult offspring under starvation. Six adult offspring (three male and three female) were randomly picked from each family. All individuals had identical larval feeding regimes, were freshly eclosed (±1 day), and were not fed as adults. Adult offspring were kept individually in 1.5-mL Eppendorf tubes with air holes in the lid and provided with filter paper disks. Survival was then recorded over 10 weeks. Adult pronotum widths were recorded for another six offspring (three male and three female) per family using a digital imaging system (QImaging, Surrey, BC, Canada) and the public domain ImageJ software (National Institute of Health, Bethesda, MD). We used pronotum width as a measure of body size because this character does not change with feeding status (Otti et al. 2009).

### Statistical analysis

All data were analyzed using the statistical platform R 3.1.0 (R Core Team 2013) and the packages *lme4* (Bates et al. 2011) and *languageR* (Baayen 2007). Each generation was analyzed separately as ancestral maternal population was no longer meaningful in the second generation of outbred crosses.

To test sources of variation in reproductive fitness of G_1_ crosses, the total number of eggs was fitted using linear mixed-effects models (LME) with week and treatment (inbred vs. outbred) as fixed factors and with their interaction term. To account for the repeated measure of egg numbers from the same female, and the different demographic histories of the stock populations, we included female nested within ancestral maternal population as random factors. To analyze the proportion of inviable eggs, we used the *cbind* function in R to construct a dependent variable combining inviable and viable egg number per week and clutch for fitting logistic random effects regression models with binomial distribution. *cbind* accounts for differences in total egg numbers. The effects of the fixed factors week and treatment and their interaction were investigated. Random effects of female nested within ancestral maternal population were included in the model. In both models, paternal and maternal pronotum widths were used as covariates to control for effects of parental body size on the respective dependent variable. The models were compared using likelihood ratio tests in a stepwise backwards fashion starting with the full model including the interaction. First, we removed the covariates to investigate their importance in the given model. We only kept covariates in the model if they significantly improved the model fit. This analysis was repeated for the G_2_ crosses except that ancestral maternal population as a random factor was excluded because it was no longer meaningful in the outcrossed lines.

To analyze G_2_ fitness, body size was analyzed as a response variable by fitting linear mixed-effects models using sex and treatment as fixed effects and female nested in maternal population as a random effect. For the survival analyses of adult offspring, we used the *survival* and *coxme* packages (Therneau et al. 2003) with the *coxme* function for a mixed effect Cox model. A survival response variable was constructed using the *Surv* function. Then, we fitted survival models, again using sex and treatment as fixed effects and female nested in maternal population as a random effect. The minimum adequate model was retained, after testing for interactions between factors. G_3_ individuals were analyzed in the same way as G_2_ except that the fitted random factor was reduced to female.

We report heterosis (*H*) for each fitness trait as relative performance (Sletvold et al. 2012):

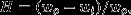
where *w*_*i*_ is the fitness trait of the inbred offspring and *w*_*o*_ is the fitness trait of the outbred offspring, with positive values indicating that outbred families outperformed inbred families, therefore heterosis. For survival, this measure was taken as the proportion of individuals surviving at the median time point of survival for each sex in each generation, respectively.

## Results

### Number of viable eggs

#### G_1_ females

**Figure 3:**
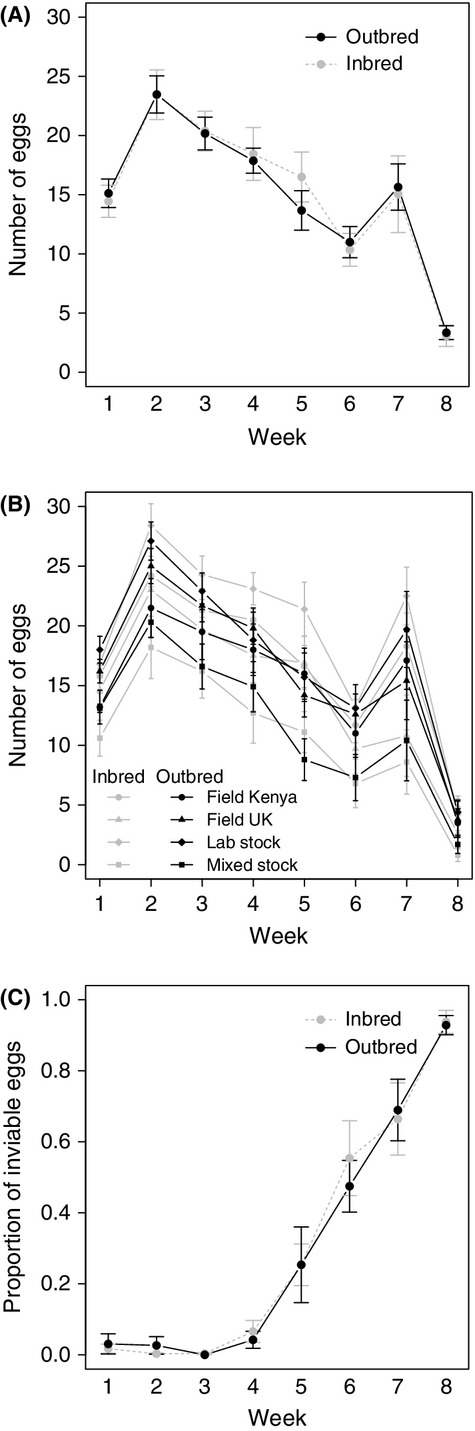
Egg number changes over time and treatment from G_1_ females. (A) total number of eggs, (B) total number of eggs separated by ancestral maternal population and treatment, (C) proportion of inviable eggs laid each week. Error bars represent one standard error. There was no significant effect of treatment on egg number.

In G_1_ females, the total egg number laid per week decreased significantly and in a similar fashion in both treatment groups (inbred or outbred) (Fig.3A,B: likelihood ratio test: week: *χ*^2^ = 237.58, *P*-value < 0.001; treatment × week: *χ*^2^ = 0.01, *P*-value = 0.98). Treatment had no effect on total egg number (Fig.3A: likelihood ratio test: *χ*^2^ = 0.19, *P*-value = 0.66). While maternal body size had a significant effect on the total number of eggs laid by G_1_ females (likelihood ratio test: *χ*^2^ = 10.20, *P-*value < 0.01), paternal body size did not (likelihood ratio test: *χ*^2^ = 1.43, *P-*value = 0.23). Over the course of the laying period, the proportion of inviable eggs increased significantly (Fig.3C: likelihood ratio test: *χ*^2^ = 4509.01, *P*-value < 0.001). Parental body size and treatment had no effect on the proportion of inviable eggs laid by G_1_ females (Fig.3C: likelihood ratio test: female pronotum width *χ*^2^ = 2.40, *P-*value = 0.12; male pronotum width *χ*^2^ = 3.07, *P-*value = 0.08; treatment *χ*^2^ = 0.08, *P*-value = 0.78). A significant interaction between treatment and week was found for the proportion of inviable eggs (likelihood ratio test: *χ*^2^ = 51.54, *P*-value < 0.001).

#### G_2_ females

**Figure 4:**
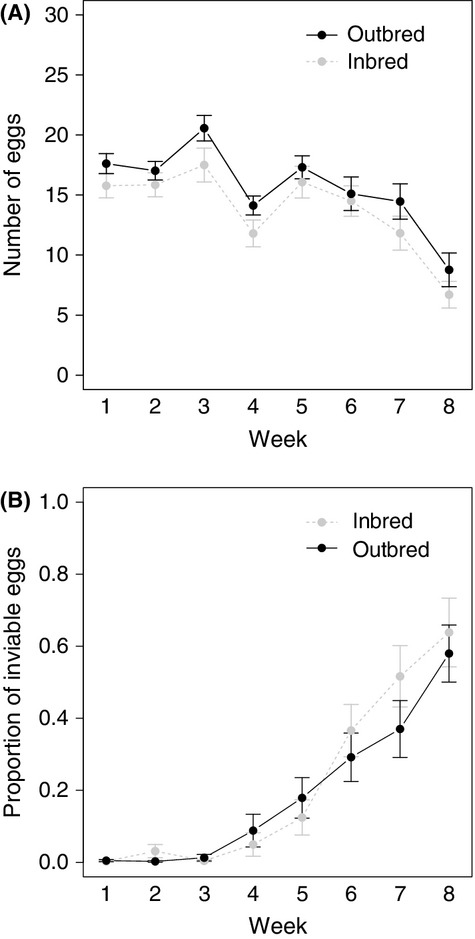
Egg number changes over time and treatment for G_2_ females. (A) total number of eggs, (B) proportion of inviable eggs laid each week.

As in G_1_ individuals, weekly egg numbers decreased significantly over the laying period, (Fig.4A: likelihood ratio test: *χ*^2^ = 98.56, *P*-value < 0.001) with no differences between treatments (Fig.4A: likelihood ratio test: *χ*^2^ = 3.05, *P*-value = 0.08), and no interaction of treatment and week (likelihood ratio test: *χ*^2^ = 0.03, *P*-value = 0.86). In contrast to G_1_ females, paternal body size had a significant effect on egg number laid by G_2_ females (likelihood ratio test: *χ*^2^ = 50.80, *P-*value < 0.001), whereas maternal body size did not (likelihood ratio test: *χ*^2^ = 0.28, *P-*value = 0.60). As in G_1_ females, the proportion of inviable eggs laid by G_2_ females increased significantly over the laying period (Fig.4B: likelihood ratio test: *χ*^2^ = 3776.62, *P*-value < 0.001), with treatment (likelihood ratio test: *χ*^2^ = 0.65, *P*-value = 0.42), paternal body size (likelihood ratio test: *χ*^2^ = 0.12, *P-*value = 0.73), and the interaction between treatment and week (likelihood ratio test: *χ*^2^ = 0.02, *P*-value = 0.89) having no effect on the proportion of inviable eggs laid. However, in G_2_ females, there was a significant effect of maternal body size on weekly proportion of inviable eggs laid (likelihood ratio test: *χ*^2^ = 4.30, *P-*value < 0.05). All outbred crosses, except one in the G_1_ crosses and three in the G_2_ crosses, produced viable eggs.

### Offspring fitness measures

**Figure 5:**
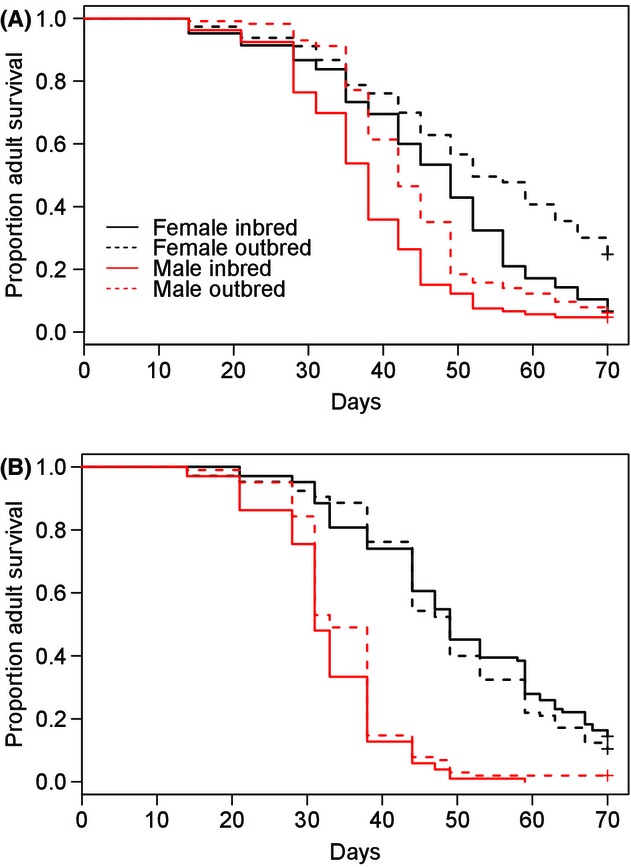
Survival analysis of (A) G_2_ adults (B) G_3_ adults under starvation. Lines represent proportion of males (red) and females (black) surviving at each sampled time point. Inbred individuals (continuous lines) had significantly reduced survival compared to outbred individuals (dashed lines).

In G_2_ individuals, outbreeding significantly increased bed bug survival under starvation (Fig.5A: Cox mixed effects model: *z* = −3.40, *N* = 438, *P*-value < 0.001). There was also a highly significant effect of sex, with survival reduced in males compared to females (Cox mixed effects model: *z* = 7.34, *N* = 438, *P*-value < 0.0001). In G_3_ individuals, males still had significantly higher mortality than females (Cox mixed effects model: *z* = 11.46, *N* = 413, *P*-value < 0.001), but there was no longer a significant mortality difference between treatments (Fig.5B: Cox mixed effects model: *z* = −0.55, *N* = 413, *P*-value = 0.58). A comparison between the generations suggests that this was due to a reduction in outbred survival (Table2).

**Table 2:** Summary of heterosis (*H*) for body size and survival under starvation in G_2_ and G_3_ adults (mean ± standard error)

	Trait	Inbred	Outbred	*H*
G_2_	Mean female pronotum width in mm	1.60 ± 0.01	1.62 ± 0.01	0.011
	Mean male pronotum width in mm	1.55 ± 0.01	1.56 ± 0.01	0.007
	Median proportion of survival	0.40 ± 0.04	0.59 ± 0.04	0.322
G_3_	Mean female pronotum width in mm	1.63 ± 0.01	1.67 ± 0.01	0.024
	Mean male pronotum width in mm	1.56 ± 0.01	1.57 ± 0.01	0.005
	Median proportion of survival	0.40 ± 0.04	0.46 ± 0.04	0.130

In both generations, females were significantly larger than males (Table2: likelihood ratio test: G_2_: *χ*^2^ = 57.12, *P*-value < 0.001; G_3_: *χ*^2^ = 108.699, *P*-value < 0.001). In G_2_, treatment had no effect on either female or male body size (likelihood ratio test: treatment *χ*^2^ = 1.81, *P*-value = 0.18; sex × treatment *χ*^2^ = 0.24, *P*-value = 0.62), whereas in G_3_, inbreeding led to the production of significantly smaller females, but not males (Table2: likelihood ratio test: treatment *χ*^2^ = 3.14, *P*-value = 0.08; sex x treatment *χ*^2^ = 4.36, *P*-value < 0.04). However, these body size differences are unlikely to explain the treatment differences in survival between G_2_ and G_3_ because females and males were larger in G_3_ than G_2_ (Table2).

Of all fitness traits measured, the most substantial heterosis was found in survival under starvation and all *H* values were positive, indicating higher fitness in outbred families (Table2).

## Discussion

Using a design that mimicked reproduction with naturally occurring gene flow between infestations by passive dispersal, and inbreeding within infestations, we found significant variation in the effects of outbreeding across fitness traits and generations. Heterosis has been shown to increase with environmental stress (Armbruster et al. 1997; Armbruster and Reed 2005), and we found that under starvation outbred individuals had significantly lower mortality, particularly after the first generation of outbreeding (*H *=* *0.322). Heterosis is predicted to be high in small populations, even in the presence of moderate gene flow (Whitlock et al. 2000), and to increase with population structure (Theodorou and Couvet 2002; Whitlock 2003). This is due to populations reaching a mutation–selection–migration balance, which can lead to sufficient among-population variance for dominance to mask the effect of recessive deleterious alleles when populations are crossed (Whitlock et al. 2000).

The highly structured nature of bed bug populations (Fountain et al. 2014) meant that two different predictions could have been made about the effects of outcrossing between geographically separated populations. Firstly, there would be heterosis, as crosses would result in introduction of novel alleles (e.g., Ebert 2002; Gaggiotti et al. 2004; Haag et al. 2005); which we observed after the first generation. Alternatively, we may have expected outbreeding depression due to loss of local adaptation, or genetic incompatibilities between populations (Lynch 1991; Whitlock et al. 2013). While we did not see immediate evidence of outbreeding depression, it is possible that the negative fitness consequences of outbreeding were not expressed until the second generation of outbreeding. The magnitude of heterosis in survival appeared reduced in G_3_ individuals (*H *=* *0.130) compared to G_2_ (*H *=* *0.322). While the median proportional survival remained the same between G_2_ and G_3_ inbred individuals, G_3_ outbred survival saw a reduction of 21.8% (Table2); therefore, the reduction in heterosis is possibly due to a decrease in outbred fitness. Reduced heterosis after repeated outbreeding corresponds to observations across several taxa that the effect of heterosis is often followed by outbreeding depression with succeeding generations of outbreeding (e.g., Edmands 1999; Fenster and Galloway 2000, 2001; Marshall and Spalton 2000; Marr et al. 2002; Whitlock et al. 2013) and suggests any beneficial effect of heterozygosity may be partly offset in subsequent generations by a breakdown in coadaptation. The deleterious consequences of outbreeding may only be expressed in the *F*_2_ offspring where recombination and segregation start to break apart positive epistatic interactions (Lynch 1991). One explanation of why we did not see more severe evidence of outbreeding depression is that this loss of fitness may also be caused by hybrid incompatibility (e.g., Dobzhansky-Muller incompatibilities) and loss of local adaptation (Charlesworth and Willis 2009). These latter two hypotheses are unlikely in our design. Each outbred G_3_ was generated by a cross to an unrelated population and so should be largely heterozygous, reducing the likelihood of recessive Dobzhansky–Muller incompatibilities (Charlesworth and Willis 2009). All stock populations have been under the same laboratory conditions over multiple generations reducing any effects of local adaptation in the first place. Therefore, the loss of positive epistasis is the most parsimonious explanation for any reduction in outbred performance in our design.

Generally, inbreeding is expected to reduce the effect of heterosis as detrimental alleles are purged from the population (Theodorou and Couvet 2002; Glémin 2003; Zhou et al. 2010). In metapopulations, there has been evidence of partial purging of deleterious mutations. Haikola et al. (2001) compared continuous and fragmented populations of *Melitaea cinxia* and found more severe inbreeding depression in continuous rather than fragmented populations. Fragmented populations are likely to experience higher levels of inbreeding due to a reduction in connectivity, so have a high potential to experience purging. Despite this, fragmented populations still experienced significant inbreeding depression, suggesting deleterious alleles were only partially purged. This partial purging reflects observed patterns in the present study. The laboratory culturing of stock populations makes it likely that individuals frequently mate with close relatives, potentially purging deleterious alleles. However, we still detected significant heterosis in outbred lines suggesting some deleterious alleles were likely fixed in the population. An alternative possibility is that heterosis was caused by overdominant loci, although this is not thought to be the primary mechanism of heterosis (Tallmon et al. 2004; Lippman and Zamir 2007; Charlesworth and Willis 2009). Metapopulation dynamics have been shown in some cases to homogenize genetic diversity across a species range (e.g., Settepani et al. 2014). This is predicted with high extinction rates relative to the influence of genetic drift within demes (Slatkin 1977). If between-population variance is low, the effects of outbreeding would likely be small or absent, which potentially could explain the small and transient positive effect of outbreeding observed here. However, while genetic information is not yet available for the stock populations used in this study, previous studies have shown that while there is low genetic diversity within demes in bed bugs, there is strong genetic differentiation between geographically close demes (e.g. Booth et al. 2012; Fountain et al. 2014). This makes the hypothesis that low outbreeding effects are due to genetic homogeneity unlikely in bed bugs. Future work should tease these hypotheses apart in this system to uncover the mechanisms behind our results, particularly by a quantification of the genetic load, and the relatedness of the stock populations. This is particularly important as the different histories of the populations are likely to have resulted in variation in purging and thus genetic load.

While it has previously been thought that bed bugs are resilient to inbreeding as large infestations can be founded by as little as a single-mated female (Fountain et al. 2014), it has not been known how outbreeding may influence bed bug ecology. We have shown that outbreeding may contribute to, at least initially, an increased likelihood in successful bed bug population establishment, or even increased dispersal. The increased starvation resistance of outbred offspring compared to inbred offspring would increase the time individuals could survive without a host at perhaps the most critical stage of successful founding. It could also facilitate increased dispersal distances between feeding, potentially leading to the spread of infestations to neighboring properties. This result is particularly relevant as one hypothesis to the bed bugs dramatic resurgence is that the increased connectivity between populations through an increase in global travel has led to a rise in multiple introductions into the same building, with the subsequent heterosis through outcrossing increasing population growth (Reinhardt 2012).

## Conclusions and future work

The focus of this study was to test how two natural colonization scenarios affect the population dynamics of founding bed bug infestations. Here, we have shown a significant beneficial effect of outbreeding in the common bed bug, whose populations are highly structured. However, this benefit was short lived, and after a further generation could no longer be detected. The results suggest that successful bed bug infestations may be rapidly established by highly inbred offspring, as well as by offspring with genetically distinct parents.

While we focused on two likely models of colonization future work should look into additional scenarios. For example, in natural bed bug populations that are associated with bat colonies (Reinhardt and Siva-Jothy 2007), where population dynamics and histories are likely to have been different. Here, pest control events did not cause extinctions and dispersal events may have been more frequent leading to differential responses to inbreeding and outbreeding.

## Supporting Information

Table S1. Breakdown of G_1_ outbred crosses. Since it was not possible to perform crosses between every combination of family line, the crossing scheme was made as balanced as possible given the availability of virgin males and females and ensuring that each reciprocal cross was performed at least once.
