# Supplementary material for: Outbreeding effects in an inbreeding insect, Cimex lectularius
Source: Ecol Evol. 2014 Dec 28;5(2):409–18. doi: 10.1002/ece3.1373 (PMC4314272; doi:10.1002/ece3.1373)
Supplement: Supplementary file 1 — Table S1. Breakdown of G1 outbred crosses. Since it was not possible to perform crosses between every combination of family line, the crossing scheme was made as balanced as possible given the availability of virgin males and females and ensuring that each reciprocal cross was performed at least once. [file ece30005-0409-sd1.doc]

Supporting Information for online publication only

Manuscript:

OUTBREEDING EFFECTS IN AN INBREEDING INSECT, *CIMEX LECTULARIUS*

Toby Fountain, Roger K. Butlin, Klaus Reinhardt, Oliver Otti

**Table S1** Breakdown of G_1_ outbred crosses. Since it was not possible to perform crosses between every combination of family line, the crossing scheme was made as balanced as possible given the availability of virgin males and females and ensuring that each reciprocal cross was performed at least once.
